# Large myxoma causing cardiac arrest during surgery

**DOI:** 10.1186/s40981-015-0026-1

**Published:** 2015-12-29

**Authors:** Takuma Maeda, Ryo Sakurai, Yoshihiko Ohnishi

**Affiliations:** Department of Anesthesiology, National Cerebral and Cardiovascular Center, 5-7-1 Fujishirodai, Suita, Osaka 565-8565 Japan

**Keywords:** Myxoma, Cardiac arrest, Surgery

## Abstract

The patient was a 67-year-old woman with a history of worsening dyspnea over several months. Cardiac echocardiography showed a large, mobile left atrial myxoma. Emergency surgery was performed. Cardiac arrest occurred during repositioning of the heart to cannulate the inferior vena cava and transesophageal echocardiography revealed the large myxoma obstructing the left ventricle. Cardiopulmonary bypass was initiated and spontaneous heartbeat returned shortly afterward. Changing myxoma position and sudden mitral orifice obstruction must be considered in these cases and once the diagnosis is made, patients should be operated on as early as possible.

## Correspondence/Findings

Large myxomas can obstruct the mitral valve and lead to sudden death [1]. We report a patient with a large left atrial myxoma that caused cardiac arrest during extirpation surgery.

**Fig. 1:**
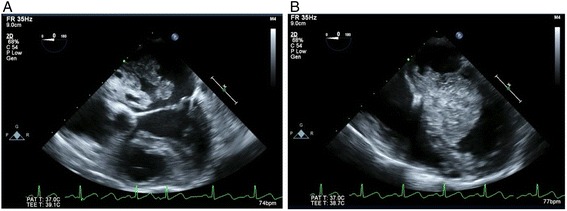
Four-chamber transesophageal echocardiogram of the large left atrial myxoma during systole (**a**) and diastole (**b**)

The patient was a 67-year-old woman with a history of worsening dyspnea over several months. The patient showed no limitations regarding her activities of daily living. Cardiac echocardiography showed a large, mobile left atrial myxoma measuring 32 mm × 44 mm moving from the left atrium to the left ventricle during the cardiac cycle (Fig. 1). The mitral orifice was impinged by the myxoma, with a mean gradient across the mitral valve of 10 mm Hg. She was transferred to our hospital for emergency surgery. Fortunately, the patient’s blood pressure was stable, ranging from 105/60 mmHg to 90/50 mmHg, her heart rate was 90–100 beats per minute, and SpO2 was 100 % with 6-L/minute inspired oxygen.

**Fig. 2:**
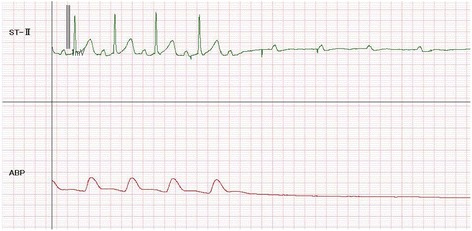
Sudden asystolic cardiac arrest with residual P wave was identified by electrocardiography when the heart was repositioned

A right radial arterial line was inserted before inducing anesthesia. Because the myxoma was causing mitral stenosis, to prevent hypotension after induction, we injected phenylephrine at 1 μg/kg/min, which stabilized her vital signs and limited blood pressure changes to no more than a 10-mmHg decrease. Aortic and superior vena caval cannulas were inserted once anticoagulation using unfractionated heparin was sufficient. Cardiac arrest occurred during repositioning of the heart to cannulate the inferior vena cava (Fig. 2) and transesophageal echocardiography revealed the large myxoma obstructing the left ventricle. The patient’s core body temperature was 36.1 °C. Cardiopulmonary bypass was initiated and spontaneous heartbeat returned shortly afterward. The duration of cardiac arrest was approximately 10 seconds. The myxoma was successfully excised and the patient was discharged without complications.

Large left atrial myxomas have caused complete obstruction of the mitral valve orifice, resulting in sudden death [1]. Simply changing body position can vary the extent of valvular obstruction [2]. Changing myxoma position and sudden mitral orifice obstruction must be considered in these cases and once the diagnosis is made, patients should be operated on as early as possible. The patient in our case developed cardiac arrest soon after aortic and superior vena caval cannulas were inserted. Therefore, cardiopulmonary bypass could be established immediately, thereby resulting in the rapid recovery of blood flow to the coronary artery and a spontaneous heartbeat. Because cardiac arrest can happen at any time, stand-by percutaneous cardiopulmonary support is ideal.
